# High prevalence of occult left ventricular diastolic dysfunction detected by exercise stress test in systemic sclerosis

**DOI:** 10.1038/s41598-022-06400-7

**Published:** 2022-02-14

**Authors:** Takato Mohri, Ayumi Goda, Kaori Takeuchi, Hanako Kikuchi, Takumi Inami, Takashi Kohno, Konomi Sakata, Kyoko Soejima, Toru Satoh

**Affiliations:** Department of Cardiovascular Medicine, Kyorin University Hospital, 6-20-2 Shinkawa, Mitaka, Tokyo 181-8611 Japan

**Keywords:** Heart failure, Vascular diseases, Systemic sclerosis

## Abstract

Despite the poor prognosis of systemic sclerosis (SSc) due to the co-occurrence of left ventricular diastolic dysfunction (LVDD), presence of occult LVDD has not been sufficiently investigated. This retrospective study aimed to reveal the prevalence and determinants of occult LVDD in patients with SSc by exercise stress test. Forty-five SSc patients (age, 63 ± 13 years; men/women, 6/39) with normal pulmonary artery pressure and pulmonary artery wedge pressure (PAWP) at rest underwent a symptom-limited exercise test with right heart catheterization using a supine cycle ergometer; haemodynamic parameters at rest, leg raise and during exercise were evaluated. Occult LVDD defined PAWP ≥ 25 mmHg during exercise was seen in 13 patients (29%). Higher PAWP, lower pulmonary vascular resistance and diastolic pulmonary pressure gradient, larger left atrium at rest, and higher PAWP during leg raise (15 ± 4 vs 10 ± 4 mmHg in non-LVDD group, p < 0.001) were observed in the occult LVDD group. The area under the ROC curve for PAWP after leg raise was largest at 0.83 (95% CI: 0.70–0.95, p = 0.001). About one-third (29%) of SSc patients with normal haemodynamics at rest showed occult LVDD. A higher PAWP after leg raise could be useful for detecting occult LVDD.

## Introduction

Pulmonary hypertension (PH) is recognised as a critical complication that occurs in approximately 7–12% of patients with systemic sclerosis (SSc)^1–3^. This condition may be caused by pre-capillary microvascular narrowing in pulmonary arterial hypertension (PAH), interstitial lung disease (ILD), veno-occlusive disease, post-capillary pulmonary hypertension due to left heart disease, or combinations of these abnormalities^4^. PH patients with SSc have a threefold higher risk of death and poorer response to therapy than those with idiopathic PAH ^5,6^. The poor prognosis of PH in patients with SSc partly results from overt left ventricular diastolic dysfunction (LVDD)^7,8^, which is frequently associated with myocardial fibrosis^9,10^. PAH-specific vasodilator drugs may have deleterious effects or may be ineffective if administered to patients with LVDD. Thus, accurate differentiation between pre-capillary and post-capillary PH is essential for risk stratification and appropriate selection of treatment.

Differentiation between pre-capillary and post-capillary PH is based on resting pulmonary arterial wedge pressure (PAWP) > 15 mmHg as the current criterion for diagnosing PH due to LVDD^11^. Some patients with LVDD may have normal resting PAWP but an abnormal increase in PAWP in response to fluid loading. In one study, several PAH patients were reclassified as occult post-capillary PH after fluid loading, which was indicative of the difficulty of differentiating PH etiology in SSc patients^12^.

The exercise stress test has been reported to facilitate early detection of PH, allowing early intervention and the opportunity to improve outcomes^13–15^. Cardiopulmonary exercise testing is useful for characterization of multifactorial exercise limitation and identification of SSc-related complications ^16^. Recently, elevated PAWP during exercise, or exercise-induced post-capillary PH, was recognised as an indicator of early heart failure with preserved ejection fraction (HFpEF)^17^, and exercise stress in the early detection of LVDD has received much attention. In addition, attempts to detect LVDD by simple methods, such as leg raise and fluid challenge, have been reported^18^. In SSc patients with normal resting haemodynamics, an inordinate increase in pulmonary artery pressure (PAP) during exercise is thought to be an early sign of pulmonary vasculopathy, as well as post-capillary pulmonary hypertension (i.e., LVDD)^19^. Hager et al. also reported that exercise-induced LVDD was common^20^. In SSc, where there is a multifactorial limitation of exercise, it is desirable to use a simple method for validation to detect occult LVDD. Furthermore, therapeutic interventions for patients with a resting mean PAP of 21–24 mmHg, called borderline PH, were recently established, and the importance of early differentiation between pre-capillary and post-capillary PH was emphasized. Therefore, we investigated the prevalence of occult LVDD in patients with SSc who were referred for clinical assessment because of dyspnoea during exertion via the exercise stress test with normal haemodynamics at rest. We also examined the factors predicting PAWP elevation during exercise among the resting haemodynamic parameters at rest and during leg raise.

## Results

### Baseline characteristics

**Figure 1:**
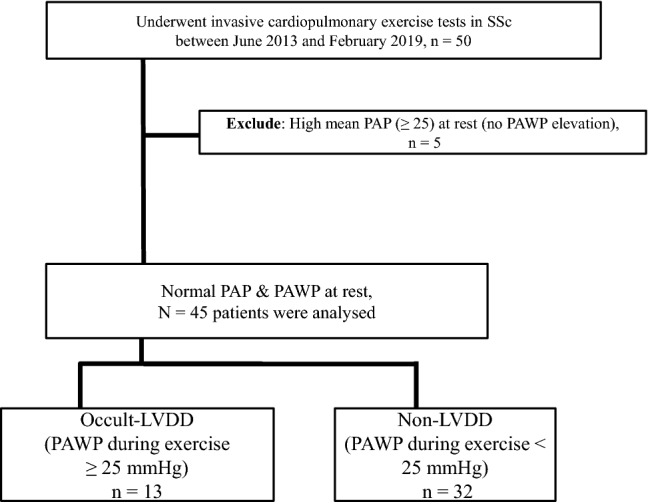
Flow chart of patients with SSc who underwent cardiopulmonary exercise testing with right heart catheterization. LVDD, left ventricular diastolic dysfunction; PAP, pulmonary artery pressure; PAWP, pulmonary artery wedge pressure; PH, pulmonary hypertension; SSc, systemic sclerosis.

**Figure 2:**
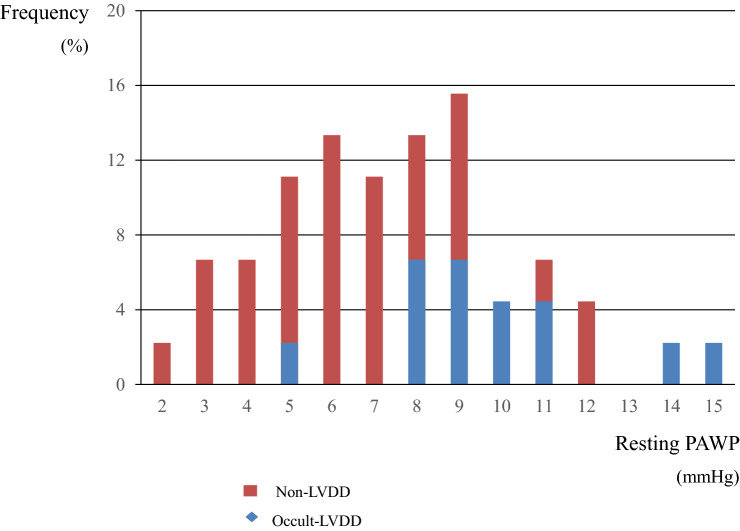
Distributions of the resting PAWP and exercise PAWP elevation. LVDD, left ventricular diastolic dysfunction; PAWP, pulmonary artery wedge pressure.

The patient flow chart is shown in Fig. 1. Among the 50 patients who underwent exercise testing, five were excluded due to a high mean PAP. No ECG changes were observed during exercise. Eventually, 45 patients (age, 63 ± 13 years; men/women, 6/39) were analysed. Of these, 13 (29%) showed occult LVDD. The distributions of the PAWP at rest by group are shown in Fig. 2. The baseline characteristics of the study groups classified by the presence or absence of occult LVDD are shown in Table 1. Parameters determined through RHC at rest were as follows: mPAP, 17 ± 4 mmHg; PAWP, 7 ± 3 mmHg; pulmonary vascular resistance (PVR), 2.3 ± 1.1 Wood units; and CO, 4.5 ± 1.4 L/min. There were no significant differences in sex, comorbidities, pulmonary function, mean PAP, CO, and 6-min walk distance (6MWD) between the two groups. Age, brain natriuretic peptide (BNP) value, and PAWP (10 ± 3 vs 7 ± 3 mmHg, p < 0.001) were significantly higher in the occult LVDD group. PVR (1.7 ± 0.8 vs 2.5 ± 1.1, p = 0.020) and diastolic pulmonary pressure gradient (DPG) (− 2 ± 2 vs 1 ± 4, p = 0.004) were significantly lower in the occult LVDD group than in the non-LVDD group. Co-morbidities, excluding intestinal lung disease, tended to be higher in the occult LVDD group. The proportion of participants with 3 or more co-morbidities was significantly greater in the occult LVDD group. There were no significant differences in the levels of autoantibodies, including anti-nuclear, anti-centromere, and anti-SCL70, between the 2 groups.

**Table 1:** Baseline characteristics of the study patients.

	Occult-LVDD (n = 13)	Non-LVDD (n = 32)	P value
Age, years	68 ± 9	60 ± 14	0.023
Sex, male/female	2/11	4/28	0.567
BMI, kg/m2	22.7 ± 2.2	21.9 ± 4.1	0.924
Duration from diagnosis, (years)	4 (2–8)	8 (3–14)	0.179
6-min walk distance, (m)	409 (380–473)	358 (280–474)	0.280
**Co-morbidities**			
Hypertension, n, (%)	7, (54)	12, (38)	0.341
Hyperlipidaemia, n, (%)	3, (23)	4, (13)	0.394
Diabetes mellitus, n, (%)	2, (15)	2, (6)	0.567
Atrial fibrillation, n, (%)	2, (15)	1, (3)	0.196
Interstitial lung disease, n, (%)	2, (15)	9, (28)	0.311
Number of co-morbidities			
0, n, (%)	5, (38)	12, (38)	0.952
1, n, (%)	4, (31)	14, (43)	0.420
2, n, (%)	1, (8)	5, (16)	0.478
3 or more, n, (%)	3, (23)	1, (3)	0.003
**Blood analysis**			
BNP, pg/dL	65 (41–161)	28 (17–70)	0.008
Hemoglobin, g/dL	11.6 ± 1.8	12.8 ± 1.3	0.016
Autoantibodies (n = 39)			
Anti-nuclear + , n, (%)	9, (75)	24, (83)	0.523
Anti-centromere + , n, (%)	8, (67)	13, (45)	0.440
Anti-SCL70 + , n, (%)	1, (8)	4, (14)	0.961
**Pulmonary function (n = 37)**			
FEV1% predicted	87 ± 19	83 ± 17	0.561
FVC % predicted	91 ± 23	89 ± 20	0.765
DLCO % predicted	63 ± 24	60 ± 18	0.678
DLCO/VA % predicted	75 ± 29	69 ± 23	0.517
%FVC/%DLCO	1.7 ± 1.1	1.6 ± 0.6	0.741
**Hemodynamic parameter (flat position)**			
Mean RAP, mmHg	4 ± 2	3 ± 2	0.076
Systolic PAP, mmHg	30 ± 6	31 ± 7	0.646
Diastolic PAP, mmHg	8 ± 3	8 ± 4	0.898
Mean PAP, mmHg	18 ± 4	17 ± 4	0.618
PAWP, mmHg	10 ± 3	7 ± 3	< 0.001
TPG, mmHg	8 ± 4	11 ± 4	0.045
DPG, mmHg	− 2 ± 2	1 ± 4	0.004
SaO_2_, %	95 ± 2	96 ± 2	0.317
SvO_2_, %	72 ± 4	73 ± 6	0.448
Cardiac output, L/min	4.8 ± 1.1	4.5 ± 1.5	0.479
PVR, Wood units	1.7 ± 0.8	2.5 ± 1.1	0.020
PAC, ml/mmHg	3.2 ± 0.6	2.9 ± 1.2	0.186
RC-time, sec	0.41 ± 0.13	0.55 ± 0.22	0.470

Values are reported as means ± standard deviation (SD), or medians (25th, 75th interquartile ranges), where appropriate.

BW, body weight; DPG, diastolic pulmonary pressure gradient; LVDD, left ventricular diastolic dysfunction; PAC, pulmonary artery compliance, PAP, pulmonary artery pressure; PAWP, pulmonary artery wedge pressure; PVR, pulmonary vascular resistance; RAP, right atrium pressure; RC time, the time constant of the pulmonary circulation, SaO_2_, O_2_ saturation in arterial blood; SvO_2_, O_2_ saturation in the pulmonary artery; TPG, transpulmonary pressure gradient; %FEV1: forced expiratory volume in 1 s %predicted, %FVC: forced vital capacity %predicted, %DLCO: diffusing capacity of the lung for carbon monoxide %predicted; DLCO/VA % predicted: diffusing capacity divided by the alveolar volume.

### Haemodynamic parameters during exercise

**Figure 3:**
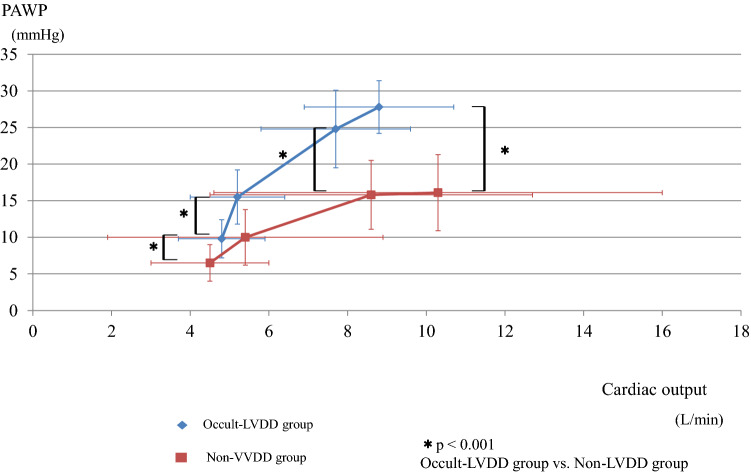
Relationships of the cardiac output to PAWP. LVDD, left ventricular diastolic dysfunction; PAWP, pulmonary artery wedge pressure.

Cardiopulmonary exercise testing with RHC findings at rest (after leg raise), AT, and peak exercise are shown in Table 2. Despite the PAWP level at rest within normal limits in both groups, its level in leg raise was significantly higher in the occult LVDD group than in the other group (15 ± 4 vs 10 ± 4 mmHg, p < 0.001). The relationships of PAWP with CO in each group are shown in Fig. 3. The mean PAWP in the occult LVDD group during exercise was higher (AT: 25 ± 5 vs 16 ± 5 mmHg, p < 0.001; peak: 28 ± 4 vs 16 ± 5 mmHg, p < 0.001), compared to PAWP in the non-LVDD group. The time constant of pulmonary circulation (RC-time) at peak exercise was significantly lower (0.23 ± 0.10 vs 0.35 ± 0.15 s, p = 0.008) in the occult LVDD group than in the non-LVDD group. CO during exercise was not significantly different between the two groups. CO at peak exercise and peak VO_2_ in our study patients were 9.9 ± 4.9 L/min and 14.3 ± 8.8 mL/min/kg, respectively, indicating that their exercise capacities were low.

**Table 2:** Haemodynamic Parameters at rest (after leg raise), anaerobic threshold, and peak exercise.

	Occult-LVDD (n = 13)	Non-LVDD (n = 32)	P value
**Rest after leg raise**			
HR, bpm	69 ± 10	74 ± 14	0.251
Systolic PAP, mmHg	40 ± 9	34 ± 8	0.027
Diastolic PAP, mmHg	13 ± 5	9 ± 4	0.012
Mean PAP, mmHg	24 ± 6	21 ± 5	0.121
PAWP, mmHg	15 ± 4	10 ± 4	< 0.001
ΔPAWP (pre-leg raise), mmHg	6 ± 2	3 ± 4	< 0.001
Cardiac output, L/min	5.2 ± 1.2	5.4 ± 3.5	0.882
PVR, wood units	1.7 ± 0.8	2.5 ± 1.3	0.014
PAC, ml/mmHg	2.9 ± 1.2	3.3 ± 3.0	0.924
RC-time, s	0.39 ± 0.21	0.51 ± 0.25	0.222
SaO_2,_ %	96 ± 1	96 ± 3	0.801
SvO_2,_ %	70 ± 4	71 ± 6	0.473
VO_2_, mL/min	211 ± 49	204 ± 70	0.764
VCO_2_, mL/min	173 ± 43	173 ± 57	0.997
R	0.81 ± 0.06	0.84 ± 0.07	0.148
VE, L/min	8.3 ± 2.7	8.1 ± 2.6	0.850
VE/VO_2_	39.9 ± 10.2	42.2 ± 11.3	0.537
VE/VCO_2_	49.2 ± 10.8	49.9 ± 11.4	0.852
**Anaerobic threshold**			
Work rate, W	28 ± 19	29 ± 22	0.837
HR, bpm	97 ± 17	104 ± 21	0.357
Systolic PAP, mmHg	56 ± 11	54 ± 12	0.478
Diastolic PAP, mmHg	17 ± 5	15 ± 5	0.189
Mean PAP, mmHg	37 ± 7	33 ± 8	0.199
PAWP, mmHg	25 ± 5	16 ± 5	< 0.001
Cardiac output, L/min	7.7 ± 1.9	8.6 ± 4.1	0.372
PVR, wood units	1.6 ± 0.8	2.4 ± 1.2	0.016
PAC, ml/mmHg	2.2 ± 0.8	2.2 ± 0.9	0.733
RC-time, s	0.25 ± 0.08	0.37 ± 0.13	0.017
SaO_2_, %	97 ± 3	95 ± 4	0.371
SvO_2_, %	52 ± 4	54 ± 7	0.277
VO_2_, mL/min	528 ± 153	598 ± 332	0.493
VCO_2_, mL/min	502 ± 142	358 ± 323	0.552
R	0.98 ± 0.14	0.98 ± 0.14	0.971
VE, L/min	20.3 ± 6.6	20.1 ± 8.4	0.935
VE/VO_2_	40.9 ± 16.3	39.9 ± 12.5	0.838
VE/VCO_2_	42.8 ± 12.6	42.1 ± 11.4	0.846
**Peak**			
Work rate, W	54 ± 24	60 ± 32	0.585
HR, bpm	108 ± 23	117 ± 26	0.318
Systolic PAP, mmHg	63 ± 9	58 ± 13	0.189
Diastolic PAP, mmHg	17 ± 7	17 ± 7	0.835
Mean PAP, mmHg	41 ± 7	36 ± 9	0.068
PAWP, mmHg	28 ± 4	16 ± 5	< 0.001
Cardiac output, L/min	8.8 ± 1.9	10.3 ± 5.7	0.359
PVR, wood units	1.6 ± 0.7	2.2 ± 1.2	0.086
PAC, ml/mmHg	1.9 ± 0.5	2.2 ± 1.0	0573
RC-time, s	0.23 ± 0.10	0.35 ± 0.15	0.008
SaO_2_, %	96 ± 4	95 ± 5	0.463
SvO_2_, %	48 ± 8	49 ± 8	0.637
VO_2_, mL/min	654 ± 205	823 ± 613	0.340
VCO_2_, mL/min	716 ± 232	924 ± 773	0.349
R	1.10 ± 0.16	1.09 ± 0.15	0.779
VE, L/min	31.0 ± 9.7	33.4 ± 19.4	0.671
VE/VO_2_	49.4 ± 18.6	44.7 ± 12.6	0.328
VE/VCO_2_	44.8 ± 12.3	41.0 ± 10.7	0.305
Peak VO_2,_ mL/kg/min	12.4 ± 3.9	15.0 ± 10.2	0.365
VE vs. VCO_2_ slope	38.2 ± 14.4	38.2 ± 21.6	0.997
PAWP-CO slope	4.1 ± 3.0	1.8 ± 1.3	0.084

Values are reported as mean ± standard deviation (SD).

BP, blood pressure; CO, cardiac output; HR, heart rate; LVDD, left ventricular diastolic dysfunction; PAC, pulmonary artery compliance, PAP, pulmonary artery pressure; PAWP, pulmonary artery wedge pressure; PVR, pulmonary vascular resistance; RC time, the time constant of the pulmonary circulation, SaO_2_, O_2_ saturation in arterial blood; SvO_2_, O_2_ saturation in the pulmonary artery; VO_2_, oxygen consumption; VCO_2_, carbon dioxide output; R, respiratory exchange ratio; VE, minute ventilation.

### Echocardiographic parameters

The echocardiographic parameters between each group are shown in Table 3. There were no significant differences between the two groups in E/e’ and E/A. Left ventricular (LV) index (112 ± 45 vs 90 ± 21 g/m^2^, p = 0.043), left atrial diameter (LAD) (39 ± 7 vs 33 ± 5 mm, p = 0.005), and left atrial volume index (LAVi) (53 ± 20 vs 39 ± 13 mL/m^2^, p = 0.033) in the occult LVDD group were significantly greater than in the non-LVDD group. HFA-PEF and H_2_FPEF scores were not significantly different between the 2 groups.

**Table 3:** Echocardiographic parameters.

	Occult-LVDD (n = 13)	Non-LVDD (n = 32)	P value
LVEF, %	69 ± 6	65 ± 5	0.018
LV mass index, g/m^2^	112 ± 45	90 ± 21	0.043
LAD, mm	39 ± 7	33 ± 5	0.005
LAVi, mL/m^2^	53 ± 20	39 ± 13	0.033
E, cm/s	74.2 ± 24.3	67.1 ± 17.0	0.088
A, cm/s	87.4 ± 19.7	72.6 ± 16.3	0.016
E/A	0.9 ± 0.2	1.0 ± 0.3	0.367
DcT, cm/s	193 ± 47	206 ± 47	0.419
E’ septal, cm/s	6.9 ± 2.1	6.9 ± 2.5	0.965
E/e’ septal, cm/s	12.1 ± 3.9	10.7 ± 3.9	0.282
E/e’ mean, cm/s	11.25 ± 3.36	9.2 ± 3.0	0.664
TAPSE, mm	24 ± 4	22 ± 3	0.165
RV FAC, %	42 ± 9	40 ± 8	0.085
HFA-PEFF score	4 (2–6)	3 (2–4)	0.451
H_2_FPEF score	2 (2–4)	2 (1–3)	0.840

Values are reported as mean ± standard deviation (SD) or medians (25th, 75th interquartile ranges), where appropriate.

E/e’ mean as average in respective value of septal and lateral E/e’.

DcT, deceleration time; LAD, left atrial diameter; LAVi, left atrium volume index; LVDD, left ventricular diastolic dysfunction; LVEF, left ventricular ejection fraction; LV mass index, left ventricular mass index; RV FAC, right ventricular fractional area change; TAPSE, tricuspid annular plane systolic excursion.

### Detection of occult LVDD

**Figure 4:**
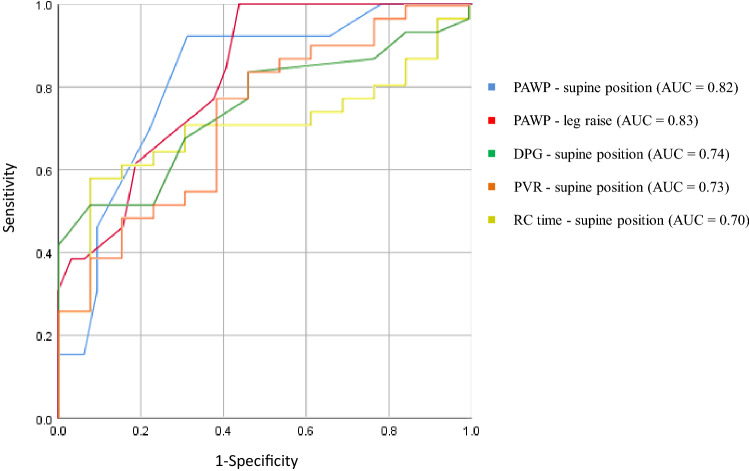
Receiver operating characteristic curves of PAWP at supine position, PAWP at leg raise, DPG at supine position, PVR at supine position and RC-time at supine position. AUC, area under the curve; DPG, diastolic pulmonary pressure gradient; PVR, pulmonary vascular resistance; RC time, pulmonary circulation time constant, PAWP, pulmonary artery wedge pressure .

Among the resting haemodynamic parameters, the ROC curve revealed that PAWP at leg raise (AUC = 0.83 [95% confidence interval (CI): 0.70–0.95], p = 0.001) and PAWP in the resting state (AUC 0.82 [95% CI: 0.69–0.95], p = 0.001) may be useful in detecting occult LVDD, followed by DPG at rest (AUC = 0.74 [95% CI: 0.60–0.89], p = 0.011), PVR at rest (AUC 0.73 [95% CI 0.57–0.89], p = 0.019), and RC time at rest (AUC = 0.70 [95% CI: 0.544–0.855], p = 0.012) (Fig. 4). A PAWP at leg raise > 11 mmHg had 100% sensitivity and 56% specificity in predicting increased PAWP ≥ 25 mmHg by exercise.

## Discussion

In this cohort of SSc patients, who were mostly female (87%) and referred because of dyspnoea on exertion, one-third of the patients with normal haemodynamics at rest showed occult LVDD via exercise test. Higher PAWP after leg raise was a useful parameter for the detection of occult LVDD in these patients.

PH is recognised as a critical complication which worsens prognosis in patients with SSc. This prognosis is mainly linked to cardiovascular damage^21^. Currently, there are no established therapeutic interventions for improving SSc prognosis. Although the clinical course and mortality in PAH have improved due to progresses in PAH-specific therapy, SSc-PH outcomes have not improved^22^. SSc-PH is not only caused by vasculopathy, but also causes diverse and sometimes overlapping mechanisms, including extensive vasculopathy, inflammation, autoimmunity, and widespread fibrosis^4,10^. SSc has the pathological hallmark of myocardial fibrosis and has been reported in 50–80% of necropsy cases^23^. Mechanistically, it appears that disease-related loss-of-function of small coronary arteries and arterioles leads to ischaemia and reperfusion injury, driving cardiac fibrotic tissue formation^24^. Furthermore, LV dysfunction gradually progresses to pulmonary arteriopathy and RV dysfunction^4,25^. In a previous study, at baseline, 17% of patients with SSc were diagnosed with overt LVDD evaluated by echocardiography at rest and associated with high mortality^7^. Concomitant pulmonary vascular disease and LVDD likely contribute to poor survival in patients with SSc-PH^8^. In cases of LV dysfunction, aggressive pulmonary vasodilator therapy by preload increase is somewhat counterproductive. Accurate differentiation between pre-capillary and post-capillary PH is important.

The clinical importance of pulmonary haemodynamics during exercise has been recognised in the early detection of PH^15,26–28^. In SSc patients, a previous study showed that exercise PH was associated with increased mortality compared to that of normal exercise haemodynamics^29,30^. In SSc, it has been noted that borderline PH (21–24 mmHg of mean PAP) has a high likelihood of developing into overt PH^31,32^; hence, attention has focussed on early interventions for managing borderline PH, and lowering the criteria has been advocated^33^. Exercise PH may be useful in predicting the future development of patients with borderline PH. Some patients with LVDD may have normal resting PAWP but show an abnormal increase in PAWP in response to fluid loading. PAWP 15 mmHg has been used, with high sensitivity, to distinguish between patients between pre-capillary and post-capillary PH; however, normal PAWP, especially between 12 and 15 mmHg, does not rule out the presence of LVDD^34^. Volume or exercise challenge during RHC may be useful to unmask the presence of LVDD. Fox et al. reported that several PAH patients were reclassified as having occult post-capillary PH after fluid loading, which was indicative of the difficulty of differentiation in patients with SSc^12^. It is known that PAWP in patients with SSc is more likely to be elevated than that of controls and other types of PH in fluid loading, and it is likely that occult LVDD is more common^35^.

It has been reported that 32% of SSc patients with normal resting haemodynamics and increased estimated pulmonary pressure on an exercise echocardiogram (> 40 mmHg) showed occult LVDD defined as increasing PAWP > 20 mmHg at exercise RHC with 1-pound weights^20^. Stamm et al. showed that about 12% of SSc patients without resting PH were classified under exercise post-capillary PH^29^. With an exercise PAWP ≥ 20 mmHg, 12 of 17 patients were reported having exercise-induced LVDD^19^. They also reported that LA dilatation might indicate occult LVDD. Our study revealed that the prevalence of occult LVDD was about one-third of SSc patients, with an exercise PAWP ≥ 25 mmHg. LA dilatation in patients with occult LVDD was also observed in our study.

Haemodynamic measurements during exercise are useful for detecting early stage LVDD, but some procedural problems of examination time, invasiveness, and ergometer equipment can occur.

Fluid challenge has been reported useful in differentiating post-capillary PH^12,36^. Among HFpEF patients with normal haemodynamics at rest, a PAWP > 15 mmHg during leg raise can indicate a PAWP ≥ 25 mmHg during exercise with 91% probability^18^. These reports show that increased preload could detect left heart dysfunction. We defined occult LVDD as ≥ 25 mmHg in the supine position during exercise and showed that PAWP during leg raise was significantly higher in the occult LVDD group. In SSc patients, PAWP > 11 mmHg at leg raise may be easier for distinguishing occult LVDD than previous provocation examinations. Haemodynamic measurement with leg raise is quick, can distinguish occult LVDD easily, and may be useful for early therapeutic intervention. In our study, exercise-induced PAWP elevation was common in patients with upper limit of normal PAWP at rest. This finding is also consistent with past recommendations^34^.

Fluid challenge is an easy manoeuvre that can be performed in any catheterization laboratory without specialised equipment. Leg raise might be even easier and safer than the fluid challenge.

This study had several limitations. Our study was a retrospective, single-centre, and non-randomized trial. Our study included a limited number of cases from a single institution, and the study population did not include overt LVDD. Although we evaluated patients with normal haemodynamics at rest, BNP levels were high in both groups, and 6MWD and peak VO_2_ showed low exercise capacity, which was different from that of healthy subjects. Our cohort was exclusively older and comprised females; therefore, the findings of this study may not be generalisable to younger adults or males. Age is an important confounder of the pulmonary vascular response and PAWP during exercise. Differences between the occult LVDD and non-LVDD groups may be owing to differences in age ^37^. There was a gap of up to 3 months between the RHC and the echocardiographic assessment, which may have affected the results. Finally, patients with pulmonary and cardiac diseases (e.g., intestinal lung disease and atrial fibrillation) were included in the cohort.

In conclusion, a high prevalence of occult LVDD was demonstrated via RHC with exercise test in SSc with normal haemodynamics at rest. The evaluation of PAWP with leg raise is easy and useful for detecting occult LVDD.

## Methods

### Study participants

Consecutive patients with SSc who exhibited dyspnoea during exertion and suspected PH underwent cardiopulmonary exercise testing (CPX) with right heart catheterization (RHC) at our hospital between June 2013 and February 2020 were eligible for inclusion in the study. Patients with a high mean PAP (≥ 25 mmHg) and PAWP elevations (> 15 mmHg) at rest were excluded (Fig. 1).

This study was approved by the Committee for Clinical Studies and Ethics of Kyorin University School of Medicine. The purposes and risks of the study were explained to the patients, who provided informed consent prior to participating. All methods were performed in accordance with the relevant guidelines and regulations.

### Right heart catheterization and cardiopulmonary exercise testing

RHC was performed with a 6-F double-lumen, balloon-tipped, flow-directed Swan–Ganz catheter (Harmac Medical Products, Inc., Buffalo, NY, USA) via a transjugular approach. Baseline haemodynamic data were recorded, the zero-reference level (mid-chest) was adjusted at the start of pressure measurement, and the PAWP was obtained as the mean value of the arterial trace during occlusion. Measurements were obtained at the end of normal expirations with patients in the supine flat position to assess the right chamber, pulmonary artery pressure (mean PAP, systolic PAP, and diastolic PAP), and PAWP^34^.

An incremental symptom-limited exercise test was performed in the supine position, with an electromagnetically braked cycle ergometer (Nuclear Imaging Table with Angio Ergometer; Lode B.V.; Groningen, Netherlands) according to the ramp protocol. During cycling, the legs were elevated to approximately 30 degrees. After the parameters were stabilised, the test consisted of a 3-min rest, followed by 3 min of warmup at an ergometer setting of 10 W (60 rpm), and, finally, testing with a 1-W increase in exercise load every 6 s (totalling 10 W/min).

During the exercise, oxygen consumption (VO_2_), carbon dioxide output (VCO_2_), and minute ventilation (VE) were measured with a metabolic cart (Cpex-1; Inter Reha Corp., Tokyo, Japan). Prior to calculating the parameters with respiratory gas analysis, an eight-point moving average of the breath-by-breath data was obtained. Peak VO_2_ was defined as the average value obtained during the last 30 s of exercise. The anaerobic threshold (AT) point was determined using the *V*-slope method, along with the following conventional criteria: VE/VO_2_ increases after decreasing or registering as flat, whereas VE/VCO_2_ remains constant or decreases^38^. The VE vs VCO_2_ slope was calculated from the start of incremental exercise to the respiratory compensation point using a least-squares linear regression^39^.

Heart rate, arterial blood pressure directly recorded in the radial artery, and 12-lead electrocardiogram were monitored continuously during the test. PAP and PAWP in the RHC were measured every minute. We used the averaged mean PAP and mean PAWP during several-second periods rather than end-expiratory measurements during exercise.

Oxygen (O_2_) saturation in arterial blood (SaO_2_) in the radial artery and O_2_ saturation in the pulmonary artery (SvO_2_) were measured at rest, AT, and peak exercise. SaO_2_ and SvO_2_ were measured simultaneously. Cardiac output (CO) was determined by the Fick method using the formula: CO (L/min) = VO_2_/ {1.34 × haemoglobin × (SaO_2_ − SvO_2_)}. The cardiac index (L/min/m^2^) was determined as follows: CO/body surface area (BSA). PVR, transpulmonary pressure gradient (TPG), DPG, pulmonary arterial compliance (PAC), and RC-time were calculated as: PVR (Wood units) = (mPAP − PAWP)/CO, TPG = mPAP − PAWP, DPG = diastolic PAP − PAWP, PAC = stroke volume/ pulse pressure, and RC-time = PVR × PAC. The slope of the PAWP-flow relationship (PAWP-CO slope) was calculated from the multipoint plots of the PAWP and CO using a least-squares linear regression ^40^. All measurements during exercise testing were performed without supplemental oxygen.

We defined SSc with LVDD as PAWP increased during the RHC exercise test and divided the patients on this basis into two groups: occult LVDD group (PAWP ≥ 25 mmHg during exercise) and non-LVDD group (PAWP < 25 mmHg during exercise).

The 6-min walk test (6MWT) was performed according to American Thoracic Society guidelines without supplemental O_2_ a day before RHC.

### Echocardiography

A transthoracic Doppler echocardiogram at the resting state was obtained and stored digitally on an Artida (Toshiba, Tokyo, Japan) or EPIQ (Philips Healthcare, Cambridge, MA USA) ultrasound system within 3 months of the RHC. Each patient was given a unique identification number to ensure that the analysis of images could be performed blinded to all invasive data and patient characteristics. The frame rate was maintained at a minimum rate of 60 frames per second. For Doppler recordings, the average of 3 to 5 consecutive beats was measured using a horizontal sweep of 75 to 100 cm/s.

LV dimensions and LAD were measured from the parasternal long axis view. The LV mass was calculated and indexed to BSA. Left ventricular ejection fraction (LVEF) was calculated using Simpson’s biplane method from the apical 4- and 2-chamber views. The left atrium (LA) maximal and LA minimal volumes were estimated from the apical 4- and 2-chamber views using biplane planimetry. LA maximal volume was measured from the frame just before the mitral valve opening, and LAVi was indexed for BSA^41^.

Mitral inflow was assessed in the apical 4-chamber view with the pulsed-wave Doppler sample volume placed at the tips of the mitral valve leaflets during diastole; the early (E) and late (A) peak diastolic velocities of the mitral inflow and the E wave deceleration time were thus measured. Mitral annular motion was assessed using pulsed-wave tissue Doppler with the sample volume placed in the septal (e’ septal). The E/e ratio was calculated^41^.

The right ventricular (RV) systolic function was assessed by measuring the tricuspid annular plane systolic excursion (TAPSE). The RV end-diastolic area (RVEDarea) and end-systolic area (RVESarea) were assessed by manual planimetry in the apical 4-chamber view, and the RV fractional area change (RVFAC) was derived using the formula: RVFAC = [(RVEDarea-RVESarea)/RVEDarea] × 100^41^.

Based on these findings, HFA-PEFF and H2FPEF scores were calculated ^42,43^.

### Statistical analysis

Continuous variables are expressed as mean ± standard deviation (SD) or median with interquartile ranges (Q1–Q3) as appropriate. Categorical variables are expressed as numbers and percentages. Group comparisons were made using Student’s t-test or Mann–Whitney U test, as appropriate, for continuous variables and χ^2^ statistics or Fisher’s exact test, as appropriate, for categorical variables. Receiver operating characteristic (ROC) curves among resting haemodynamic parameters were drawn, and the area under the curve (AUC) was calculated. The cut-off value that resulted in the highest product sensitivity and specificity was considered the best for the detection of exercise PAWP elevation. Statistical comparisons were considered significant at p < 0.05. All analyses were performed using SPSS statistical software (version 26.0; IBM Corp., Armonk, NY, USA).
